# Diagnosis of perinuclear anti-neutrophil cytoplasmic antibody-associated microscopic polyangiitis in silicotics: case report

**DOI:** 10.1186/s40557-016-0108-1

**Published:** 2016-05-14

**Authors:** Ji-Won Lee, Jun-Pyo Myong, Yeong-Jin Choi, Seyoung Lee, Bum Seak Jo, Jung-Wan Koo

**Affiliations:** Department of Occupational and Environmental Medicine Seoul St. Mary`s Hospital, College of Medicine, The Catholic University of Korea, 222 Banpo-Daero Seocho-gu, Seoul, 137-701 Republic of Korea; Department of Hospital Pathology, Seoul St. Mary`s Hospital, College of Medicine, The Catholic University of Korea, Seoul, Republic of Korea; Center for Occupational and Environmental Medicine, The Catholic University of Korea, Seoul St. Mary’s Hospital, 222 Banpo-Daero Seocho-gu, Seoul, 137-701 Republic of Korea

**Keywords:** Occupational exposure, Silicon dioxide, Silicosis, Autoimmune disease

## Abstract

**Background:**

An association between silica exposure and autoimmune diseases including rheumatoid arthritis, systemic sclerosis, systemic lupus erythematosus, and anti-neutrophil cytoplasmic autoantibody (ANCA)-associated vasculitis has been made.

**Case presentation:**

A 56-year-old male presented with silicosis and had an occupational history of precious metal processing for 30 years and a 30 pack-year smoking history. The patient was diagnosed with pneumoconiosis and received compensation. No other complications were reported for pneumoconiosis. The patient suddenly presented with a non-specific headache for several days and microscopic hematuria was identified upon examination in the outpatient clinic. Following several weeks, the patient presented with aggravated dyspnea and hemoptysis, and his Modification of Diet in Renal Disease estimated glomerular filtration rate indicated acute kidney injury. Diagnostic analysis revealed perinuclear ANCA-associated microscopic polyangiitis (p-ANCA-associated MPA).

**Conclusion:**

Exposure to silica dust was likely one of the cause of p-ANCA-associated MPA. Possible pathogenic mechanisms of autoimmune diseases in silicotics and emphasis of the necessity for early diagnosis are discussed.

## Background

Silica dust exposure and silicosis have been hypothesized to be associated with autoimmune diseases [1]. Although a complete pathogenic mechanism is yet unknown, the increasing number of cases linking silica exposure and autoimmune diseases and the dose-dependent relationship between silica exposure and risk of autoimmune diseases [2] support the aforementioned hypothesis.

Anti-neutrophil cytoplasmic autoantibody (ANCA)-associated vasculitis in silicotics is difficult to diagnose due to its non-specific clinical findings and need for pathological confirmation. Hemoptysis is a common clinical manifestation in both ANCA-associated vasculitis and simple or complicated pneumoconiosis with comorbid fungal ball infection. Furthermore, in such patients, hemoptysis may mimic pneumonia on a chest radiograph. Apart from these clinical similarities, there are several distinctions between the treatment of ANCA-associated vasculitis and pneumonia. ANCA-associated vasculitis requires immunosuppressive therapy, whereas pneumonia may be treated with antibiotics. Therefore, differential diagnosis is necessary.

Few cases of silicosis with ANCA-associated vasculitis, which is involved in both the lung and kidney, have been described. We report the case of a 56-year-old male with silicosis and perinuclear ANCA-associated microscopic polyangiitis (p-ANCA-associated MPA).

## Case presentation

A 56-year-old male had a prior history of pneumoconiosis and an occupation history of precious metal processing for the previous 30 years. The patient had received Special Examinations for Pneumoconiosis in 2005. Pneumoconiosis was compensated by the Ministry of Employment and Labor and the Korea Workers’ Compensation and Welfare Service (COMWEL); however, the official complication for pneumoconiosis was not reported. A chest radiograph showed calcified pneumoconiotic nodules and bilateral nodular type q/q opacities and profusions of 3/3 in both lung fields, and was consistent with the diagnosis of pneumoconiosis in accordance with the International Labour Office (ILO) classification [3]. The patient had a 30 pack-year history of smoking and had been treated for hypertension with nifedipine (120 mg).

The patient presented with a non-specific headache lasting for several days to the outpatient clinic of the Department of Occupational and Environmental Medicine. Blood urea nitrogen (BUN) and serum creatinine levels were 52.5 mg/dl and 4.69 mg/dl, respectively, and were elevated compared with previous values of 20.4 mg/dl for BUN and 1.16 mg/dl for serum creatinine. Microscopic hematuria was detected on urinalysis. The patient was referred to a nephrologist and a kidney sonogram was performed. Renal parenchymal disease affecting both kidneys and a left simple renal cyst of 1.2 cm in size were detected. A kidney biopsy was recommended by the nephrologist but was not undertaken due to the high medical cost.

Approximately 2 weeks following the last visit at the outpatient clinic, the patient was admitted to emergency complaining of aggravated dyspnea and massive hemoptysis. There was no history of fever, joint pain, or skin rashes.

Vital signs revealed an elevated systolic blood pressure of 230 mmHg, increased diastolic blood pressure of 160 mmHg, and a respiratory rate of 30–36 breaths per min. Initially, percutaneous oxygen saturation checked by a rescue worker was 44 %. Auscultation revealed coarse breathing sounds without wheezing throughout both lung fields. Analysis of arterial blood gases on room air showed a p*H* of 7.179, a PaCO_2_ of 36.0 mmHg, a PaO_2_ of 82.0 mmHg, a HCO_3_ of 12.9 mmol/l, and an O_2_ saturation of 92.9 %.

Laboratory findings revealed normocytic-normochromic anemia. The erythrocyte sedimentation rate (ESR) was found to be increased at 58 mm/h. Serum levels of high-sensitivity C-reactive protein (hs-CRP) and potassium were elevated (3.32 mg/dl and 7.2 mEq/l, respectively). A BUN level of 101 mg/dl and a serum creatinine level of 10.63 mg/dl were found. The Modification of Diet in Renal Disease estimated glomerular filtration rate (MDRD estimated GFR) was calculated to be 5.07 ml/min/1.73 m^2^ indicating acute kidney injury. Normal serum protein levels were detected. A routine urinalysis revealed a glucose level of 1+, more than 100 red blood cells per high power field, and proteinuria (3+).

**Fig. 1:**
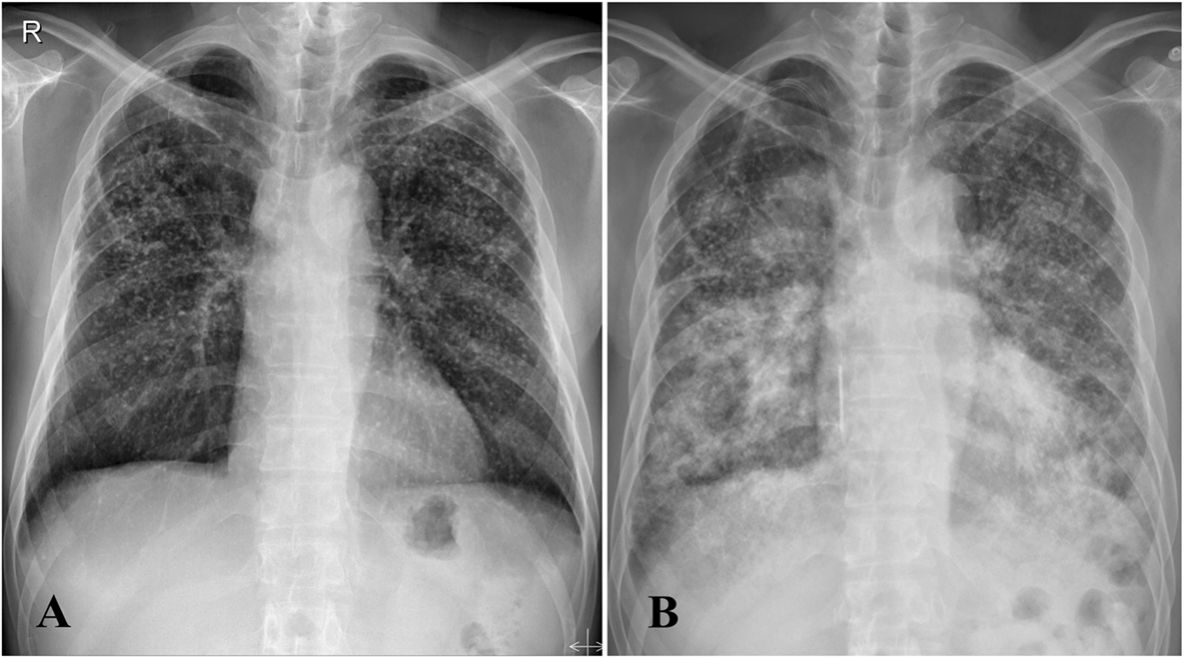
Simple chest radiographs. **a** 6 months before admission, numerous nodules in both lungs were observed. The majority of the nodules were quite dense suggesting calcification. **b** Upon admission, newly developed haziness was noted in both lungs suggesting pneumonia, edema, or hemorrhage

**Fig. 2:**
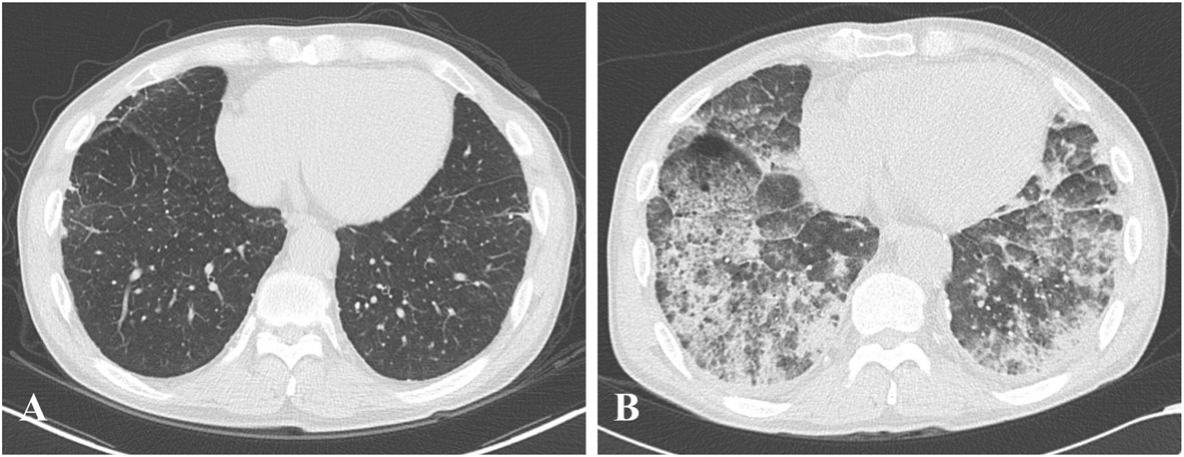
Low-dose chest computed tomography (LDCT). **a** 2 years before admission, numerous calcified nodular infiltrations were present in whole lung fields. Minimal subpleural septal thickening with reticulations in the right middle lobe (RML) and both lower lobes (BLLs) were observed. Lymph node enlargements with calcifications were found in the mediastinum, hilum, interlobar, and lobar regions. **b** Upon admission, multifocal patchy areas of consolidation were found in both lungs suggesting extensive pneumonia. Furthermore, diffuse ground glass opacity was noted in both lungs suggesting pulmonary edema with or without hemorrhage

An initial chest radiograph revealed newly developed haziness in both lungs suggesting pneumonia, pulmonary edema, or hemorrhage (Fig. 1b). Underlying pneumoconiosis was observed with little interval change. Non-enhanced abdomen and pelvis computed tomography (CT) showed two potential simple cysts in the left kidney. Low-dose chest CT revealed a newly developed multifocal patchy consolidation in both lungs indicating extensive pneumonia, and a diffuse ground glass opacity suggesting pulmonary edema with or without hemorrhage in both lungs (Fig. 2b).

**Fig. 3:**
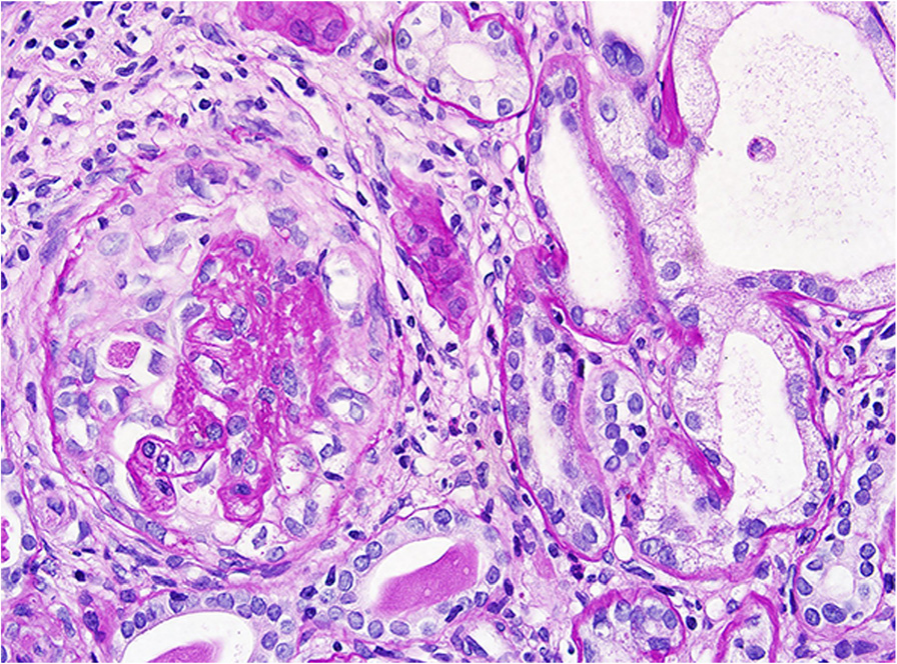
Light microscopic examination using PAS staining. Light microscopic examination was performed using special staining with M-trichrome, silver, PAS, and Verhoeff-Van Gieson elastic. Fibrocellular crescents in some glomeruli and chronic interstitial fibrosis in tubulointerstitium were observed. The kidney biopsy was found to be consistent with chronic sclerosing glomerulonephritis with acute tubular necrosis, possibly due to ANCA-associated crescentic glomerulonephritis and arteriosclerosis. The electron microscopic examination revealed totally sclerosed glomeruli which was no specific deposition for ANCA-associated crescentic glomerulonephritis

**Fig. 4:**
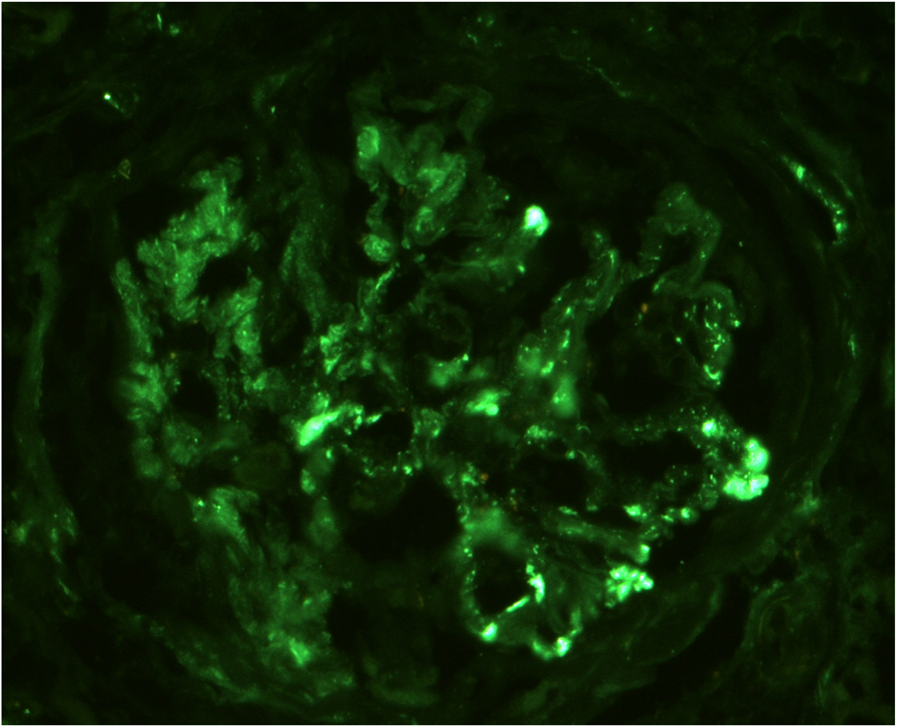
Immunoflourescence microscopic finding. Immunofluorescence revealed scant amounts of granular C3 deposits. In this examinations, no specific deposition for ANCA-associated crescentic glomerulonephritis was found

An ultrasonography guided renal biopsy was performed to investigate the cause of kidney injury and hematuria. Light microscopy revealed a chronic sclerosing glomerulonephritis suggesting ANCA-associated crescentic glomerulonephritis (Fig. 3). Both moderate acute tubular necrosis and arteriosclerosis were also observed. Electron microscopy revealed totally sclerosed glomeruli. P-ANCA was detected using indirect immunofluorescence microscopy in serum (at a titer of 1:80), and in biopsied kidney (Fig. 4).

**Fig. 5:**
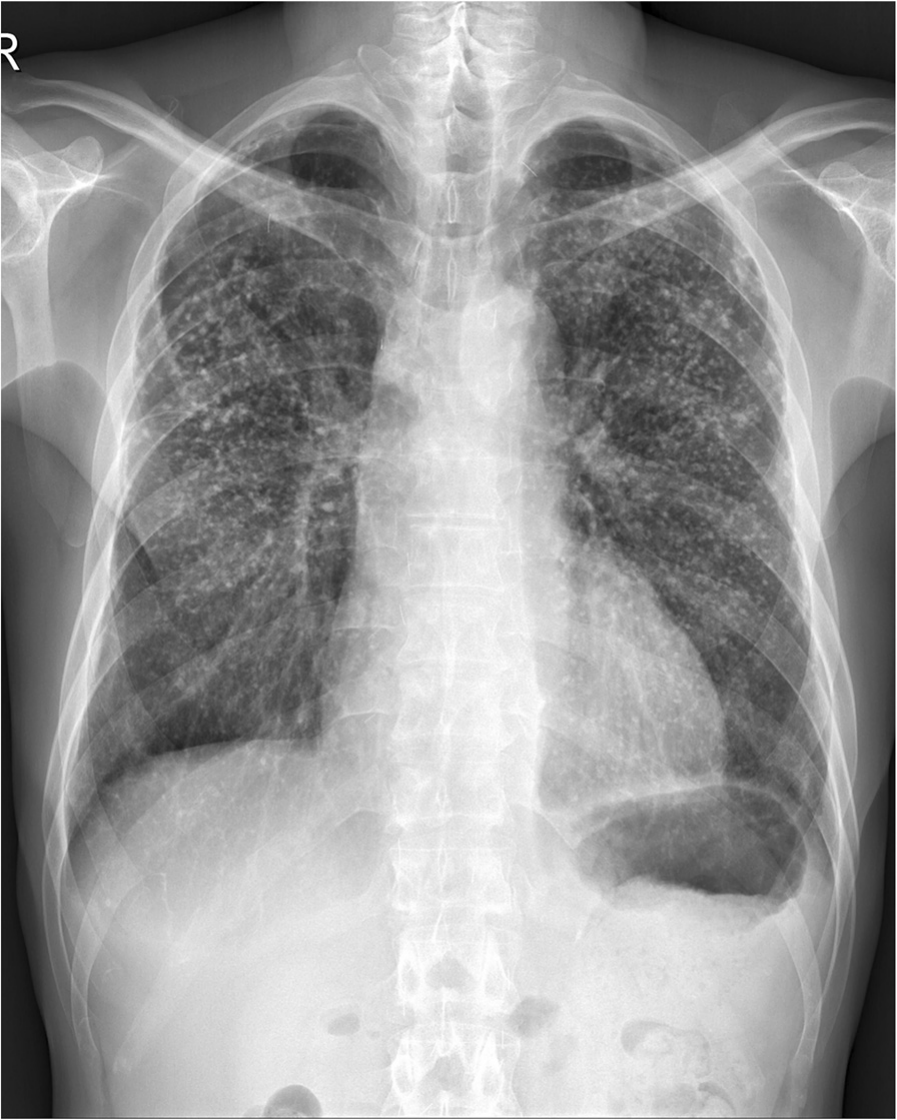
Simple chest radiograph after the remission. About 11 months after the remission, there was cleared pneumonic infiltration in both lower lung fields since prior study. Little interval change of underlying pneumoconiosis was seen

The patient was received a single course of combination therapy with intravenous cyclophosphamide 11.4 mg/kg/day and prednisolone 2 mg/kg/day for remission induction. In addition, this patient was treated with six plasma exchange sessions for removal of pathological circulating factors, such as ANCAs. After 2 weeks, his p-ANCA titer decreased as 1:20, and 2 weeks later achieved clinical remission and restored general condition. However, he remained on hemodialysis (3 sessions per week). Azathioprine (1.5 mg/kg/day) was described for 3 months as a maintenance therapy and oral prednisolone was tapered off within 2 months after discharge. By 11 months, he maintained remission without immunosuppressant (Fig. 5).

## Conclusions

We reported a case of p-ANCA-associated MPA that was diagnosed by renal biopsy in a patient with silicosis presenting with hemoptysis, hematuria, and acute kidney injury.

Silica dust exposure may be associated with an increased risk of autoimmune diseases including ANCA-associated vasculitis. Exposure to silica dust may contribute to some possible mechanisms of autoimmunity [4]. First, silica dust may play a role in the production of autoantibodies. Second, silica induces anti-Fas autoantibody production that inhibits apoptosis of responder T cells in CD4 positive peripheral blood T cells. Third, increasing Fas expression in regulatory T cells (Treg), which regulate the activation of responder T cells, leads to apoptosis of Treg. The aforementioned possible mechanisms may induce an imbalance of T cells (responder T cells > Treg cells). Finally, dysregulation of autoimmunity results in complications of autoimmune disorders [4].

In cases with multiorgan involvement of the respiratory and renal systems, infectious, malignant, and autoimmune diseases should be considered in the differential diagnosis. In the present case, both infectious and malignant conditions were excluded; therefore, autoimmune diseases such as Wegener’s granulomatosis, Goodpasture syndrome, and systemic vasculitis were considered as a possible cause [5]. Laboratory results showed increased p-ANCA levels, and pathological analysis suggested an ANCA-associated MPA. Finally, the patient was diagnosed with ANCA-associated MPA involving both the lung and kidney. As the treatment of ANCA-associated MPA involves immunosuppressive therapy and differs from that of infectious (antibiotics including anti-fungal or anti-bacterial agents) or malignant conditions (chemotherapy) [6], early diagnosis remains crucial. Fortunately, the patient presented here was discharged with a successful outcome.

ANCA-associated MPA, which is one of the most common ANCA-associated vasculitides involving both the lung and kidney, was previously diagnosed in a patient with silicosis [6]. Therefore, in cases of silicosis with presenting symptoms of hemoptysis, hematuria, proteinuria, or renal failure, an early assessment for autoimmune disease, including ANCA-associated MPA, should be carefully considered to prevent end organ damage.

## Consent

Written informed consent was obtained from the patient for publication of this case report and any accompanying images.

## Abbreviations

BUN: blood urea nitrogen
COMWEL: Ministry of Employment and Labor and the Korea Workers’ Compensation and Welfare Service
ESR: erythrocyte sedimentation rate
GFR: glomerular filtration rate
hs-CRP: high-sensitivity C-reactive protein
ILO: International Labour Office
LDCT: low-dose chest computed tomography
MDRD: Modification of Diet in Renal Disease
MPA: microscopic polyangiitis
M-trichrome: Masson trichrome
p-ANCA: perinuclear anti-neutrophil cytoplasmic autoantibody
PAS: periodic acid-Schiff stain
Treg: regulatory T cells.
